# Association of Maternal PM_2.5_ Exposure with Preterm Birth and Low Birth Weight: A Large-Scale Cohort Study in Northern Thailand (2016–2022)

**DOI:** 10.3390/toxics13040304

**Published:** 2025-04-13

**Authors:** Pak Thaichana, Patumrat Sripan, Amaraporn Rerkasem, Theera Tongsong, Suraphan Sangsawang, Sawaeng Kawichai, Worawut Srisukkham, Chanane Wanapirak, Sirinart Sirilert, Natnita Mattawanon, Chotiros Phanpong, Krongporn Ongprasert, José G. B. Derraik, Kittipan Rerkasem

**Affiliations:** 1 Office of Research Administration, Chiang Mai University, Chiang Mai 50200, Thailand; pak.th@cmu.ac.th; 2 Environmental-Occupational Health Sciences and Non-Communicable Diseases Research Center, Research Institute for Health Sciences, Chiang Mai University, Chiang Mai 50200, Thailand; amaraporn.rer@cmu.ac.th (A.R.); sawaeng.kaw@cmu.ac.th (S.K.); j.derraik@auckland.ac.nz (J.G.B.D.); 3 Research Center for Infectious Disease and Substance Use, Research Institute for Health Sciences, Chiang Mai University, Chiang Mai 50200, Thailand; patumrat.sripan@cmu.ac.th; 4 Department of Obstetrics and Gynecology, Faculty of Medicine, Chiang Mai University, Chiang Mai 50200, Thailand; theera.t@cmu.ac.th (T.T.); cwanapir@gmail.com (C.W.); sirinart.s@cmu.ac.th (S.S.); natnita.m@cmu.ac.th (N.M.); 5 Regional Health Promotion Center1 Hospital, Chiang Mai 50100, Thailand; suraphan.s@anamai.mail.go.th (S.S.); chotiros.p@anamai.mail.go.th (C.P.); 6 Department of Computer Science, Faculty of Science, Chiang Mai University, Chiang Mai 50200, Thailand; worawut.s@cmu.ac.th; 7 Department of Community Medicine, Faculty of Medicine, Chiang Mai University, Chiang Mai 50200, Thailand; krongporn.o@cmu.ac.th; 8 Department of Pediatrics: Child and Youth Health, Faculty of Medical and Health Sciences, University of Auckland, Auckland 1010, New Zealand; 9 Department of Women’s and Children’s Health, Uppsala University, 753 10 Uppsala, Sweden; 10 Clinical Surgical Research Center, Department of Surgery, Faculty of Medicine, Chiang Mai University, Chiang Mai 50200, Thailand

**Keywords:** particulate matter 2.5 exposure, preterm birth, low birth weight, small for gestational age, maternal risk factors, particulate matter 2.5 threshold levels, health risks

## Abstract

Air pollution exposure has been increasingly linked to adverse pregnancy outcomes. This study aimed to investigate the effects of PM_2.5_ exposure throughout pregnancy on preterm birth, low birth weight (LBW), and small for gestational age (SGA). We analyzed a cohort of 16,965 pregnant women living in northern Thailand between 2016 and 2022. PM_2.5_ concentration data were collected from two air quality monitoring stations operated by the Pollution Control Department (PCD) of Thailand. Logistic regression models were used to assess the association between daily PM_2.5_ exposure and pregnancy outcomes. PM_2.5_ exposure at levels exceeding 37.5 μg/m^3^ throughout pregnancy significantly increased the risk of preterm birth (aOR = 2.19, *p* < 0.001) and LBW (aOR = 1.99, *p* < 0.001) compared to the reference group (15.1–37.5 μg/m^3^). However, exposure at levels ≤15.0 μg/m^3^ also increased the risk for both outcomes compared to the same reference group. Subgroup analysis of high-risk pregnant women, including women aged > 35 years, with pre-pregnancy BMI (<18.5), pregnancy-induced hypertension, and nulliparous women, showed that the range of the critical PM_2.5_ exposure threshold was 32.3–38.4 μg/m^3^ for preterm birth and 31.2–38.2 μg/m^3^ for LBW. This study highlights the significant association between PM_2.5_ exposure and adverse pregnancy outcomes and suggests the need for targeted interventions to mitigate its effects on maternal and child health.

## 1. Introduction

Air pollution is a global health issue, particularly fine particulate matter with a diameter of 2.5 microns or less, known as PM_2.5_. Pregnant women are particularly vulnerable to PM_2.5_ exposure, which is linked to maternal inflammation [1], increased risks of preterm birth [2], and low birth weight [3]. PM_2.5_ can also affect feto-placental hemodynamics, potentially increasing umbilical arterial resistance [4]. It also contributes to adverse pregnancy outcomes, such as increased risk of fetal mortality and placental damage, through oxidative stress and mitochondrial apoptosis in trophoblasts [5].

One of the most concerning effects of PM_2.5_ exposure during pregnancy is the increased risk of premature birth, which means preterm delivery before 37 weeks [6]. Importantly, preterm delivery is a major factor associated with infant morbidity and mortality in the perinatal period and throughout the life course. Preterm infants have a high risk of developing complications such as respiratory problems, infections, and delayed development [7,8]. Several studies have shown a relationship between higher PM_2.5_ concentrations and increased preterm birth rates [2,9,10]. For example, a study in China found that an increase in PM_2.5_ per 10 µg/m^3^ is associated with an increase of 4% in the risk of preterm birth [11].

There is also evidence suggesting that exposure to high PM_2.5_ concentrations during pregnancy may increase the risk of low birth weight (LBW), defined as birth weight below 2500 g [12]. Additionally, PM_2.5_ exposure during pregnancy has been linked to small for gestational age (SGA) which is defined as birth weight below the 10th percentile for gestational age and sex. As reported, preterm birth, LBW, and SGA infants are at high risk of developing complications during childbirth and both short- and long-term adverse health outcomes, including metabolic disorders, in adulthood [3,13,14,15]. Evidence suggests that PM_2.5_ disrupts placental function and nutrient transport, which affects fetal growth [16], but the mechanism is not clearly understood.

Although numerous studies have demonstrated a relationship between PM_2.5_ exposure and adverse pregnancy outcomes, studies remain limited in Southeast Asia, and none of these studies have been conducted in the Thai population. PM_2.5_ pollution in Southeast Asia is characterized by seasonal biomass burning, industrial emissions, and transboundary haze. Studies in Indonesia and Vietnam have linked this pollution to adverse pregnancy outcomes, with exposure patterns that differ from those in Western populations [17,18].

Given that the differences in composition and magnitude of air pollution and the varying susceptibility to environmental stressors in different populations, it is crucial to investigate the potential health risks within a given local/regional context. Chiang Mai exhibits a unique pattern of PM_2.5_ sources influenced by agricultural practices and geographic factors. During the dry season, the primary sources of PM_2.5_ is the burning of local crop residue and transboundary pollution from the Thai–Myanmar border. In contrast, during the wet season, local traffic emissions become the dominant source of PM_2.5_ [19,20]. This particular seasonal variation underscores the importance of region-specific studies to better understand the impact of PM_2.5_ exposure in this population.

Therefore, the primary objective of our study was to examine the relationships between PM_2.5_ concentrations and the likelihood of adverse pregnancy outcomes, namely preterm birth, LBW, and SGA, among Thai pregnant women. Also, we aimed to identify the period during pregnancy that is most sensitive to the potential harmful effects of PM_2.5_ and to explore potential interactions between PM_2.5_ exposure and other risk factors.

Although the WHO guidelines have established that PM_2.5_ concentrations exceeding 37.6 μg/m^3^ daily and 15.1 μg/m^3^ annual [21,22] are associated with increased mortality risk in the general population, the threshold at which PM_2.5_ exposure begins to affect pregnancy outcomes remains unknown. Therefore, another objective of our study was to identify the critical threshold level of PM_2.5_ exposure at which adverse effects on pregnancy outcomes begin to occur. This is intended to contribute essential evidence for the formulation of air quality control guidelines, with particular emphasis on protecting pregnant women.

Consequently, the study of the relationship between PM_2.5_ and pregnancy outcomes are of paramount importance, both in terms of public health and medical research. These results will enhance our understanding of the effects of air pollution on the health of pregnant women and infants and lead to the development of more effective policies and preventive measures. Ultimately, this will improve the overall quality of life for the population.

## 2. Materials and Methods

### 2.1. Ethics Approval

All procedures for this study were reviewed and approved by the Institutional Review Boards of both hospitals (Maharaj Nakorn Chiang Mai Hospital: SUR-2566-0534; Regional Health Promotion Center1 Hospital Chiang Mai: 34/2567) on 6 November 2023. The requirement for written informed consent was waived by the ethics committees due to the study’s retrospective design.

### 2.2. Study Design

This study was conducted in Muang district, Chiang Mai Province, which has the highest population density in the northern part of Thailand, with a permanent population of almost 200,000. Chiang Mai is situated in northern Thailand, in a flat plain surrounded by high mountain ranges. In Chiang Mai Province, the major source of PM_2.5_ during episodes of haze has been identified as biomass burning [20]. This study aimed to assess the associations between PM_2.5_ exposure and the risk of preterm birth, low birth weight (LBW), and small gestational age (SGA). The data used in this study were derived from two hospitals, namely, Maharaj Nakorn Chiang Mai Hospital and Regional Health Promotion Center1 Hospital, Chiang Mai, which are the two largest maternal and child health hospitals in Chiang Mai Province, located 4.7 km apart from each other. Maharaj Nakorn Chiang Mai Hospital is a tertiary referral center that primarily manages patients with severe and complex conditions. In contrast, Regional Health Promotion Center 1 Hospital is a secondary care facility focused on preventive care and a broader range of maternal and child health services. To ensure that all socioeconomic levels of pregnant women are included in the study, we used data from the two major hospitals which include different insurance schemes.

Pregnant women who received antenatal care and delivered in these two hospitals between 1 January 2016, and 31 December 2022, were included in this analysis. We excluded pregnant women who experienced abortion or stillbirth during the index pregnancy, had multiple pregnancy and for whom information regarding maternal or infant health was missing. To ensure data independence and avoid bias, pregnant women with multiple deliveries at the hospital were included only once in the study. For woman with multiple eligible deliveries, only the most recent delivery was selected for inclusion in the analysis.

This study presents the categories of daily average of PM_2.5_ concentration, each reflecting different levels of potential health impacts according Thai national standards and WHO guidelines [21,22]: “very good” (≤15.0 μg/m^3^), “good and moderate” (15.1–37.5 μg/m^3^), and “starting to affect health and harmful to health” (>37.5 μg/m^3^).

### 2.3. Meteorological Data and Exposure Assessment

In this study, meteorological data were collected from hourly reports provided by the Pollution Control Department during the study period. According to the protocol of the Chiang Mai National Air Quality Monitoring Network, the concentrations of PM_2.5_ in Chiang Mai were measured consecutively at two designated national monitoring sites. These measurements utilized the tapered element oscillating microbalance (TEOM) method, with hourly readings aggregated into daily averages. From 1 January 2015 to 31 December 2022, the Yupparaj High School site (T36), the monitoring site nearest to the two study hospitals, served as the primary data source for PM_2.5_ concentrations. However, when needed, the missing data from site T36 were supplemented by measurements from the Chiang Mai Government Center site (T35) that is also located in the urban area, approximately 6 km from the two hospitals. This approach ensured comprehensive and continuous monitoring of PM_2.5_ concentrations in the area.

Daily PM_2.5_ data were imputed using the random forest machine learning method when the data were missing from both stations [23]. Concentrations of PM_2.5_ were calculated to daily average for the different periods and for the entire duration of pregnancy up to the day of delivery. To evaluate association of PM_2.5_ concentration exposure during different stages of pregnancy, which may occur in various windows of peak level of PM_2.5_ concentration and result in different pregnancy outcomes, the daily average exposure concentration of individual pregnant women was estimated for four exposure windows: first trimester (<14 weeks), second trimester (≥14 to <27 weeks), third trimester (≥27 weeks), and the entire pregnancy. This was calculated according to concentration of PM_2.5_ from the start to end date of each trimester.

Supplementary Figure S1 shows the study area in Chiang Mai City, Thailand. The detailed map includes the two air quality monitoring stations (T35 and T36, indicated by blue stars) and the two Chiang Mai hospitals where maternal and infant data were collected.

### 2.4. Data Collection and Outcome Measurements

This study aimed to create comprehensive perinatal care profiles by collecting and analyzing extensive data on infant characteristics. Baseline information was established, including sociodemographic factors and pregnancy-related complications. The study collected a wide range of maternal data (including age, Asia–Pacific classification of BMI at pre-pregnancy, occupation, education level, parity, as well as pregnancy-related information such as gestational age, number of antenatal care visits, and mode of delivery). Infant data included APGAR scores at 5 min, sex, and birth weight.

To establish a detailed timeline of pregnancy, we recorded or calculated several key dates (the end of first and second trimesters and the date of delivery). Our database contained information on preterm births (delivery before 37 weeks of gestation), LBW (birth weight < 2500 g), and SGA (birth weight below the 10th percentile for gestational age and infant sex according to INTERGROWTH-21st standards) [24].

### 2.5. Subgroup Analysis

To address the critical threshold concentration of PM_2.5_ exposure at which pregnancy outcomes begin to be impacted, subgroup analysis was performed. This analysis is specifically for pregnant women in high-risk groups, including pregnant women aged over 35 years underweight pregnant women, women experiencing pregnancy-induced hypertension, and nulliparous women. Since exposure levels ≤15 μg/m^3^ daily are considered acceptable by both Thai national standards and WHO guidelines [21,22], we focused specifically on women exposed to levels exceeding this daily threshold throughout pregnancy to examine potential adverse effects of sustained elevated exposure. Then, we excluded participants with daily PM_2.5_ exposure ≤15 μg/m^3^, classified as “very good air quality”. By concentrating on higher exposure daily levels (>15 μg/m^3^), we aim to detect and quantify the effects of 10 μg/m^3^ increment of daily average of PM_2.5_ concentration on maternal and infant outcomes in environments where air pollution may pose health risks.

### 2.6. Statistical Analysis

The characteristics of the study population, distribution of daily concentrations of PM_2.5_, and meteorological data were presented using descriptive statistics. Categorical variables were displayed as frequencies with proportions, while continuous variables were reported as medians with interquartile range (IQR) and the range of maximum and minimum values. The distribution of maternal and infant characteristics between the three groups—pregnant women exposed to PM_2.5_ ≤ 15.0 µg/m^3^, 15.1–37.5 µg/m^3^, and PM_2.5_ > 37.5 µg/m^3^—were compared using the chi-square or Fisher’s exact test for categorical characteristics and the Kruskal–Wallis test for continuous characteristics. The associations between PM_2.5_ exposure and infant outcomes (including preterm birth, LBW, and SGA) were estimated using binary logistic regression models with adjustment of potential confounding factors. The odds ratios (ORs) were adjusted for potential confounders, including maternal age, pre-pregnancy BMI, frequency of antenatal care visits, parity, pregnancy-induced hypertension, thalassemia, history of abortion, infant sex, gestational diabetes, maternal occupation, and maternal education level. Adjusted odds ratios (aORs) with 95% confidence intervals (CIs) were calculated to indicate the risk of preterm birth, LBW, and SGA for an increase in PM_2.5_. A backward stepwise selection method was employed to determine the most important factors associated with the outcomes. Variables with *p*-values greater than 0.2 were excluded from the model in each step, while those with *p*-values less than 0.05 were retained. To assess the predictive capability of PM_2.5_ for preterm birth and LBW, receiver operating characteristic (ROC) curve analysis was performed. The area under the curve (AUC) was calculated to quantify the overall predictive performance, with 95% confidence intervals (CIs) computed to estimate the precision of the AUC. The *p*-values were also calculated to determine the statistical significance of the predictive relationship. A *p*-value < 0.05 was considered statistically significant. The ROC curve analysis was performed separately for preterm birth and LBW to compare the predictive performance of PM_2.5_ for each condition.

All statistical analyses were conducted using STATA software (StataCorp. 2021. Stata Statistical Software: Release 17. StataCorp LLC, College Station, TX, USA). All the statistical analyses were calculated using two-sided tests with a 5% level of significance.

## 3. Results

### 3.1. Study Selection

**Figure 1:**
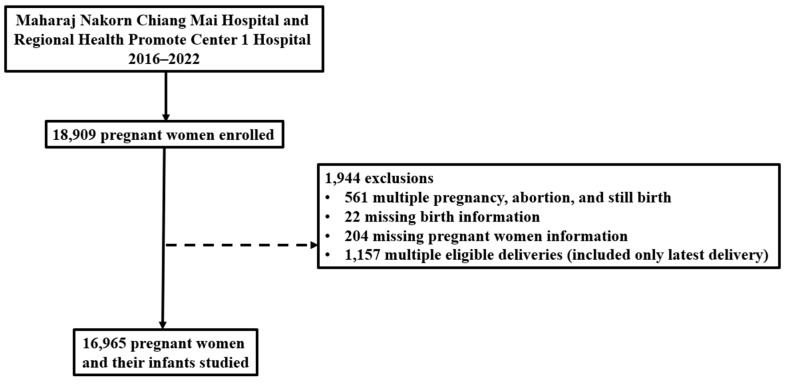
Flow diagram of participant recruitment for the study with reasons for exclusion.

Overall, 18,909 pregnant women attended antenatal clinics in the two hospitals, but 1944 failed to meet the inclusion criteria and were excluded. Thus,16,965 women and their infants were studied (Figure 1). Of those, 8420 (49.6%) women delivered at Maharaj Nakorn Chiang Mai Hospital and 8545 (50.4%) delivered at Regional Health Promotion Center1 Hospital, Chiang Mai. Daily average PM_2.5_ concentrations for each pregnancy exposure window were calculated using data from two monitoring sites of the Pollution Control Department in Chiang Mai, providing a comprehensive dataset for analyzing the relationship between PM_2.5_ exposure and pregnancy outcomes.

### 3.2. Population Characteristics

Table 1 presents the characteristics of pregnant women and infants stratified by PM_2.5_ exposure levels (≤15.0, 15.1–37.5, and >37.5 μg/m^3^). Among 16,965 pregnancies, 15,615 (92.0%) pregnant women were exposed to PM_2.5_ concentrations ≤15.0 µg/m^3^ (lowest-exposure group), 15.1–37.5 µg/m^3^ (middle-exposure group), and >37.5 µg/m^3^ (highest-exposure group), respectively, throughout pregnancy, while 1350 (8.0%) pregnant women were exposed to PM_2.5_ concentrations >37.5 µg/m^3^. Most participants aged 20–35 years (81.4%) had normal BMI (46.7%), worked indoors (52.8%), had a secondary school education or higher (81.7%), attended more than eight antenatal care visits (59.6%), and were nulliparous (53.9%). Three dominant complications during pregnancy were pregnancy-induced hypertension (PIH) (8.7%), gestational diabetes mellitus (14.2%), and thalassemia (16.9%). Most infants were male (50.8%) and born via vaginal delivery (69.6%) with APGAR scores greater than 7 (98.5%). The median gestational age at delivery was 38 weeks (IQR: 38, 39), and median birth weight was 3065 g (IQR: 2785, 3350).

Maternal characteristics were comparable among the three groups. Maternal age distribution varied across exposure groups, with a higher proportion of women aged >35 years in the lowest-exposure group (≤15.0 μg/m^3^) compared to other groups (18.4% vs. 14.4% in both other groups, *p* = 0.019). Similarly, women with obesity (BMI > 25.0 kg/m^2^) were more prevalent in the lowest-exposure group (27.2%) compared to the middle- (24.2%) and highest-exposure groups (20.9%), *p* = 0.015.

Women in the highest-exposure group (>37.5 μg/m^3^) attended significantly fewer antenatal care visits (median: seven) compared to both lower-exposure groups (median: eight for both), *p* < 0.001. Notably, the rate of pregnancy-induced hypertension (PIH) was higher in the lowest-exposure group (12.8%) compared to the middle- (8.4%) and highest-exposure groups (10.7%), *p* < 0.001. Also, gestational diabetes mellitus (GDM) was more prevalent in the lowest-exposure group (17.3%) compared to the middle- (14.0%) and highest-exposure groups (14.4%), though this difference was not statistically significant (*p* = 0.146).

The proportion of infants with APGAR scores < 7 at 5 min was significantly higher in the lowest-exposure group (4.3%) compared to the middle- (1.4%) and highest-exposure groups (2.2%), *p* < 0.001, while Cesarean section rates were highest in the lowest-exposure group (38.7%) compared to the middle- (30.1%) and highest-exposure groups (30.4%), *p* < 0.001. Additionally, women in the lowest-exposure group were more likely to receive care at Maharaj Nakorn Chiang Mai Hospital, a tertiary referral center (58.2% vs. 49.4% and 49.3%, *p* = 0.001).

### 3.3. Characteristics of PM_2.5_ Exposure with Preterm Birth and Low Birth Weight: A Pregnancy Stage Analysis

PM_2.5_ concentrations during the rainy season (June to November) were consistently low (Supplementary Figure S2). In the study area, the annual average of PM_2.5_ concentrations from 2015 to 2022 reached the level of “harmful to health” (>15 μg/m^3^) according to the Thai national standards and World Health Organization (WHO) annual PM_2.5_ air quality guideline (the red dashed line). Despite some reductions in variability, the median concentrations remained above the WHO guideline throughout the study period, underscoring the persistent problem of elevated PM_2.5_ levels in the region (Supplementary Figure S3).

As shown in Table 2, preterm births accounted for 1780 cases (10.5%), while LBW occurred in 1894 cases (11.2%). Additionally, SGA cases represented 1361 infants (8.0%). During the first trimester, median PM_2.5_ exposure was slightly higher among preterm births (22.3 μg/m^3^) compared to term births (22.0 μg/m^3^), while LBW and SGA infants had lower exposures (21.8 and 21.3 μg/m^3^) than both NBW and non-SGA infants (22.1 μg/m^3^). In the second trimester, preterm births had slightly lower median PM_2.5_ concentrations (20.2 μg/m^3^) compared to term births (20.8 μg/m^3^). LBW and SGA infants had exposure levels (20.5 and 20.7 μg/m^3^) that were highly comparably to those of both NBW and non-SGA infants (20.4 μg/m^3^). Third-trimester PM_2.5_ exposure was lower for both preterm births and LBW infants (19.4 μg/m^3^) than for term births and NBW infants (19.9 μg/m^3^). Similarly, SGA and non-SGA infants had comparable third-trimester exposures (19.9 and 19.8 μg/m^3^). When analyzing the entire pregnancy period, preterm births had a lower median PM_2.5_ exposure (26.5 μg/m^3^) compared to term births (27.1 μg/m^3^). Similarly, LBW and SGA infants had slightly lower exposures (26.9 μg/m^3^) compared to NBW and non-SGA infants, both of which had a median exposure of 27.1 μg/m^3^.

### 3.4. Factors Associated with Preterm Birth, Low Birth Weight, and Small for Gestational Age: A Univariate Logistic Regression Analysis

The associations between maternal, infant, and environmental factors with preterm birth, LBW, and SGA were analyzed using univariate logistic regression, as shown in Supplementary Table S1. Factors that significantly increased the risk of preterm birth included maternal age, with higher risk observed in pregnant women aged <20 and >35 years (OR 1.61 and 1.58, respectively, *p* < 0.001) compared to those aged 20–35 years. Additional risk factors were pre-pregnancy underweight (BMI < 18.5 kg/m^2^) (OR 1.48, *p* < 0.001) compared to normal BMI, outdoor occupation (OR 1.17, *p* = 0.011) compared to indoor occupation, primary school education or lower (OR 1.22, *p* = 0.001), and PIH (OR 1.96, *p* < 0.001). Furthermore, exposure to PM_2.5_ throughout pregnancy at levels >37.5 μg/m^3^ was significantly associated with an increased risk of preterm birth (adjusted OR = 2.46, *p* < 0.001) compared to the reference group (15.1–37.5 μg/m^3^). Also, exposure at levels ≤15.0 μg/m^3^ was linked to a higher risk (adjusted OR = 3.36, *p* < 0.001) relative to the same reference group. Conversely, the factors that significantly decreased the risk of preterm birth included adequate antenatal care (≥8 visits) (OR 0.35, *p* < 0.001), thalassemia (OR 0.55, *p* < 0.001), and multiparity (OR 0.89, *p* = 0.018).

Factors that significantly increased the risk of LBW included maternal age, with higher risk observed in pregnant women aged <20 and >35 years (OR 1.62 and 1.58, respectively, *p* < 0.001) compared to pregnant women aged 20–35 years. Additional risk factors were pre-pregnancy underweight (BMI < 18.5 kg/m^2^) (OR 1.79, *p* < 0.001) compared to normal BMI, outdoor occupation showed (OR 1.21, *p* = 0.001) compared to indoor occupation, PIH (OR 1.98, *p* < 0.001), and gestational diabetes (OR 1.21, *p* = 0.004). Furthermore, exposure to PM_2.5_ throughout pregnancy at levels >37.5 μg/m^3^ was significantly associated with an increased risk of LBW (adjusted OR = 2.17, *p* < 0.001) compared to the reference group (15.1–37.5 μg/m^3^). Also, exposure at levels ≤15.0 μg/m^3^ was linked to a higher risk (adjusted OR = 3.18, *p* < 0.001) relative to the same reference group. Conversely, factors that significantly decreased the risk of LBW included adequate antenatal care (≥8 visits) (OR 0.49, *p* < 0.001), thalassemia (OR 0.52, *p* < 0.001), male infants (OR 0.86, *p* = 0.001), and multiparity (OR 0.70, *p* < 0.001). For SGA, significant risk factors included maternal age <20 years (OR 1.40, *p* = 0.009) compared to those aged 20–35 years, pre-pregnancy underweight (BMI <18.5 kg/m^2^) (OR 1.68, *p* < 0.001), and PIH (OR 1.54, *p* < 0.001). Conversely, protective factors included higher pre-pregnancy BMI categories, with overweightness (23.0–24.9 kg/m^2^) (OR 0.74, *p* = 0.001) and obesity (>25.0 kg/m^2^) (OR 0.70, *p* < 0.001) significantly reducing the risk of SGA. Multiparity was also associated with a lower risk of SGA (OR 0.51, *p* < 0.001).

### 3.5. Association of PM_2.5_ Exposure with Preterm Birth, Low Birth Weight, and Small for Gestational Age: A Multivariable Analysis Across Pregnancy Stage

Table 3 presents the association between PM_2.5_ exposure categories during different periods of pregnancy and adverse outcomes, specifically preterm, LBW, and SGA, after adjustment for potential confounders. PM_2.5_ exposure over the entire pregnancy period at levels >37.5 μg/m^3^ was significantly associated with an increased risk of preterm birth (adjusted OR = 2.19, *p* < 0.001) compared to the reference group (15.1–37.5 μg/m^3^). Also, exposure at levels ≤15.0 μg/m^3^ was linked to a higher risk (adjusted OR = 3.18, *p* < 0.001) relative to the same reference group. For the risk of LBW, PM_2.5_ exposure over the entire pregnancy period at levels >37.5 μg/m^3^ was significantly associated with an increased risk of LBW (adjusted OR = 1.99, *p* < 0.001) compared to the reference group (15.1–37.5 μg/m^3^). Also, exposure at levels ≤15.0 μg/m^3^ was linked to a higher risk (adjusted OR = 3.02, *p* < 0.001) relative to the same reference group. However, no statistically significant association was observed between PM_2.5_ exposure and SGA.

When analyzed by trimester, no statistically significant associations were found between PM_2.5_ exposure in the first, second, or third trimesters and any of the adverse outcomes (preterm birth, LBW, or SGA).

### 3.6. Determining the Critical Threshold of PM_2.5_ Exposure for Adverse Pregnancy Outcomes Using ROC Curve Analysis

Table 4 presents the results of subgroup analyses (*n* = 16,520). Among pregnant women aged over 35 years, a significant association was observed between average PM_2.5_ exposure throughout pregnancy and LBW (adjusted OR 1.35, *p* = 0.001), indicating a 35% increased risk of LBW per 10 μg/m^3^ increase in PM_2.5_. In the subgroup of women with pre-pregnancy BMI < 18.5 kg/m^2^, PM_2.5_ exposure was significantly associated with preterm birth (adjusted OR 1.26, *p* = 0.016), suggesting a 26% higher risk of preterm birth per 10 μg/m^3^ increase in PM_2.5_. In the subgroup of women with pregnancy-induced hypertension, PM_2.5_ exposure was associated with both preterm birth (adjusted OR 1.09, *p* = 0.030) and LBW (adjusted OR 1.11, *p* = 0.012), indicating 9% and 11% increased risks, respectively, per 10 μg/m^3^ increase in PM_2.5_ exposure. For nulliparous women, PM_2.5_ exposure was significantly associated with both preterm birth (adjusted OR 1.12, *p* = 0.023) and LBW (adjusted OR 1.15, *p* = 0.022), suggesting a 12% and 15% increased risk, respectively, per 10 μg/m^3^ increase in PM_2.5_ throughout pregnancy.

**Figure 2:**
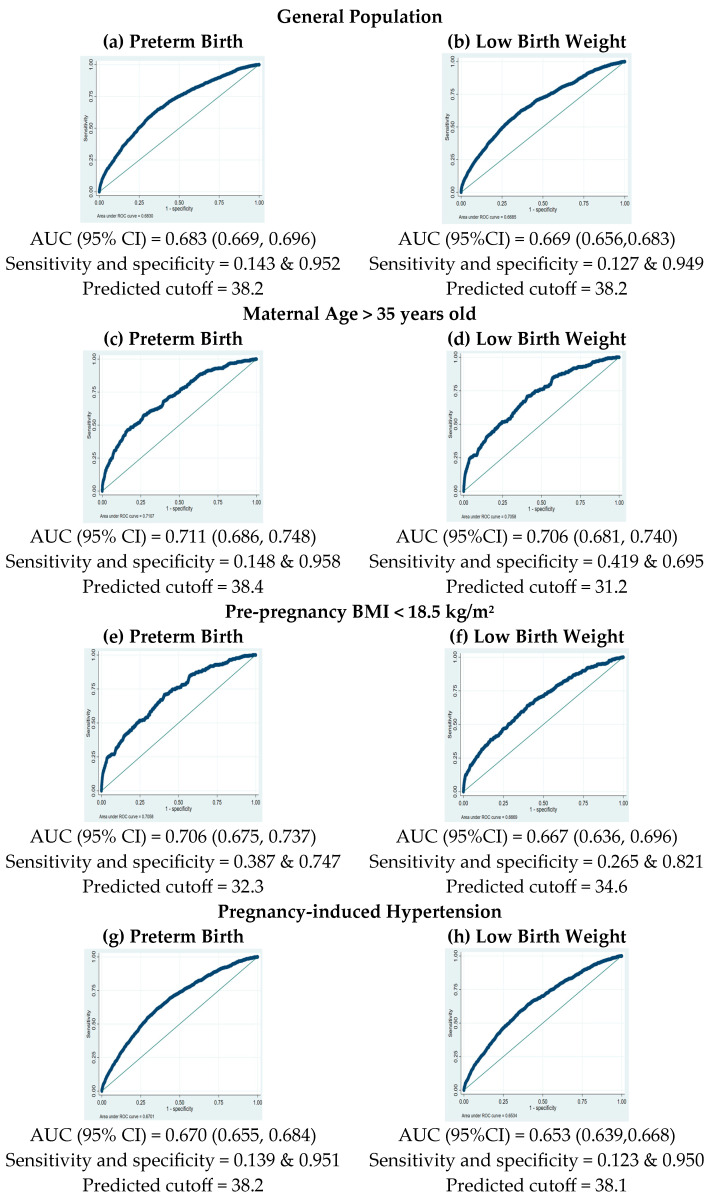

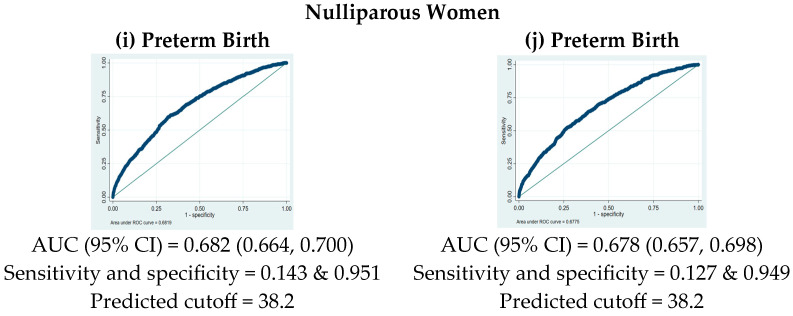
Receiver operating characteristic (ROC) curves for PM_2.5_ exposure in predicting preterm birth (left column) and low-birth-weight risks (right column) in different subgroups.

The analysis utilized ROC curves to determine the optimal PM_2.5_ cutoff concentration for classifying preterm birth and LBW, as shown in Figure 2. The PM_2.5_ cutoff concentration for both outcomes, preterm birth and LBW, was 38.2 μg/m^3^, with specificity of 95.2% and 94.9%, for preterm birth and LBW, respectively, and sensitivity of 14.3% and 12.7%, respectively. The predictive ability assessed by the area under the curve (AUC) was 68.3% (95% CI: 66.9–69.6%) for preterm birth and 66.9% (95% CI: 65.6–68.3%) for LBW. In pregnant women aged over 35 years, the PM_2.5_ cutoff value for LBW was at 31.2 μg/m^3^, with a sensitivity at 41.9% and a specificity at 69.5%. The AUC for this subgroup was 70.6% (95% CI: 68.1–74.0%). For women with pre-pregnancy BMI < 18.5 kg/m^2^, the optimal PM_2.5_ cutoff value for preterm birth was 32.3 μg/m^3^, with a sensitivity at 38.7%, specificity at 74.7%, and an AUC for this group at 70.6% (95% CI: 67.5–73.7%). In the subgroup of PIH, the PM_2.5_ cutoff value was 38.2 μg/m^3^ for preterm birth and 38.1 for LBW, with specificity at 95.1% and 95.0%, respectively, sensitivity at 13.9% and 12.3%, respectively, and AUCs for this group at 67.0% (95% CI: 65.5–68.4%) for preterm birth and 65.3% (95% CI: 63.9–66.8%) for LBW. For nulliparous women, the PM_2.5_ cutoff value was 38.2 μg/m^3^ for preterm birth and LBW, with specificity at 95.1% and 94.9%, respectively, sensitivity at 14.3% and 12.7%, respectively, and AUCs for this group at 68.2% (95% CI: 66.4–70.0%) for preterm birth and 67.8% (95% CI: 65.7–69.8%) for LBW, respectively.

## 4. Discussion

The primary finding of this study is that PM_2.5_ exposure during pregnancy is an independent risk factor for preterm birth and low birth weight (LBW) among Thai pregnant women, even after controlling for potential confounding factors. The finding is further supported by the observation of significantly reduced gestational age and birth weight among infants born to exposed mothers. Notably, the effect size of this association is particularly pronounced among women aged over 35 years, those with a pre-pregnancy BMI of less than 18.5 kg/m^2^, and those complicated with pregnancy-induced hypertension.

To the best of our knowledge, this study represents the first comprehensive investigation of PM_2.5_ exposure effects on pregnancy outcomes in the Thai population, where unique seasonal patterns of air pollution and biomass burning may influence exposure–outcome relationships differently than previously studied populations. Our findings of an increased risk of adverse pregnancy outcomes align with those reported in a previous study involving a Chinese population [11], a large study conducted in Africa [25], and recent meta-analyses encompassing diverse populations worldwide [26,27], underscoring the global impact of air pollution on pregnancy outcomes. Although no prior studies in Thailand have specifically examined the effects of PM_2.5_ on pregnancy outcomes, a large nationwide study of pregnant women found that exposure to PM_10_ throughout pregnancy was associated with reduced birth weight [28], indirectly supporting our finding regarding the adverse effects of air pollution on pregnancy outcomes.

In terms of the timing of PM_2.5_ exposure during pregnancy and its effects, results vary across studies. Previous research has reported significant associations between PM_2.5_ exposure during individual trimesters and adverse pregnancy outcomes. Some studies found associations with preterm birth and LBW throughout pregnancy [29], while others reported significant relationships only during the first, second, and third trimesters [18]. In our study, PM_2.5_ exposure throughout the entire pregnancy at levels >37.5 μg/m^3^ was significantly associated with an increased risk of preterm birth and LBW compared to the reference group (15.1–37.5 μg/m^3^). However, exposure at levels ≤15.0 μg/m^3^ was also associated with a higher risk for both outcomes relative to the same reference group. This controversy may be attributable to the higher proportion of complications observed in the group of mothers exposed to low PM_2.5_ levels (≤15.0 μg/m^3^), which may be associated with a greater proportion of women in the tertiary hospital compared to the other groups. In our study, we considered the complications of pregnant women, including GDM, PIH, and thalassemia, in the adjusted model. However, other complications that influence preterm birth and LBW may not have been considered due to the lack of data. Furthermore, no significant association with SGA was found, and individual trimester exposures did not show significant relationships with pregnancy outcomes. These findings align with several studies that reported similar associations between sustained PM_2.5_ exposure and preterm birth and LBW [26]. Our findings on the association between PM_2.5_ exposure and adverse pregnancy outcomes align with those of previous studies. A meta-analysis by Sun et al. demonstrated that PM_2.5_ exposure throughout pregnancy is linked to an increased risk of preterm birth [30]. Similarly, large cohort studies have reported associations between PM_2.5_ and reduced birth weight across various countries [31,32]. However, the effect size observed in our study differs, possibly due to the PM_2.5_ levels in our population, our focus on entire pregnancy exposure, and unique geographic factors. Methodological differences in data collection and analysis may also contribute to the variance in effect magnitude. Our results differ from studies that found significant trimester-specific effects that may relate to the components of PM_2.5_ [33,34] and those reporting associations with SGA [14]. The discrepancy in SGA findings may be verified using differences in population characteristics, exposure methods, or regional PM_2.5_ composition. A meta-analysis and other studies have found that PM_2.5_ exposure during pregnancy increased the risk of SGA, particularly with first, second or third trimester exposure [35,36], but other studies reported no significant relationship with SGA, emphasizing the need for standardized methods and more nuanced analyses of exposure timing. In this population, exposure to PM_2.5_ did not have a significant effect on SGA, suggesting that PM_2.5_ did not directly disrupt fetal growth, leading to LBW. Rather, the higher risk of LBW in our study group seems to be largely influenced by an increased likelihood of preterm birth, as preterm deliveries are associated with lower birth weight. In addition, genetic and nutritional factors may play larger roles on SGA than PM_2.5_ exposure. Interestingly, cumulative PM_2.5_ exposure throughout pregnancy was significantly associated with adverse pregnancy outcomes, whereas trimester-specific exposure was not. This suggests that sustained exposure may be more critical than short-term peaks [37,38].

Subgroup analyses revealed variability in the impact of PM_2.5_, with heightened risk observed among pregnant women aged over 35 years (aOR 1.35 for LBW) and those with pre-pregnancy BMI < 18.5 kg/m^2^ (aOR 1.26 for preterm birth). These findings underscore the compounding effects of maternal risk factors and environmental exposures, emphasizing the need for targeted interventions and enhanced prenatal care in areas with poor air quality [39,40]. The association between PM_2.5_ exposure and PIH was particularly notable, as increased risks for preterm birth and LBW in PIH cases suggest a potential mechanistic link between air pollution, maternal cardiovascular health, and infant outcomes [41]. Among nulliparous women, higher PM_2.5_ concentrations were associated with an increased risk of preterm birth and LBW in our study, whereas this association was not observed in the Korean population [42]. The ROC curve analysis showed moderate predictive ability of PM_2.5_ for adverse pregnancy outcomes (AUC of 68.3% for preterm birth and 66.9% for LBW), with improved performance in the general population. The identified threshold of 31.2 μg/m^3^ for LBW in pregnant women aged over 35 years. A threshold of 32.3 μg/m^3^ was observed for preterm birth in mothers with a pre-pregnancy BMI < 18.5, while thresholds of 38.1 and 38.2 μg/m^3^ were associated with preterm birth and LBW in pregnant women with PIH. Similarly, a threshold of 38.2 μg/m^3^ was associated with both preterm birth and LBW in nulliparous women. These findings emphasize the need for targeted interventions to reduce PM_2.5_ exposure among vulnerable groups. Public health strategies should include raising awareness about the risks of outdoor PM_2.5_ exposure during pregnancy [43]. Additionally, the threshold of 37.5 µg/m^3^ for PM_2.5_ exposure is a significant finding for air quality management efforts aimed at protecting maternal and infant health. This threshold corresponds to the concentration at which health risks begin for the general population, as recommended by the WHO [22].

The mechanisms by which PM_2.5_ exposure impacts pregnancy outcomes are complex. PM_2.5_ can induce systemic inflammation, which affects fetal growth [44], and cause oxidative stress, damaging cells and DNA [45,46]. It can also disrupt endocrine function by altering hormone levels critical for pregnancy [47]. Furthermore, PM_2.5_ may influence gene expression through epigenetic mechanisms [48,49]. PM_2.5_ exposure may also impair placental development and function, reducing oxygen and nutrient transport to the fetus. This could exacerbate the risks of preterm birth and LBW, particularly in vulnerable subgroups, and alter the microbiome, contributing to adverse outcomes [50].

The seasonal variation in PM_2.5_ concentrations in Chiang Mai, peaking during the dry season (December to May), underscores the need for season-specific interventions. This pattern, consistent with findings from studies conducted in regions with seasonal air quality changes, suggests that pregnancy planning and protective measures during high-pollution periods may be beneficial. The peak concentrations observed in March highlight the importance of targeted interventions during high-risk times, especially in areas with distinct wet and dry seasons [51]. Our findings suggest that pregnant women should avoid outdoor work, particularly during high-pollution periods; this is especially important, as we found that outdoor work was associated with both preterm birth and LBW. This recommendation extends beyond PM_2.5_ exposure, as other pollutants such as PM_10_, carbon monoxide, ozone, and additional components of PM_2.5_ have also been linked to an increased risk of preterm birth and/or LBW [28,52,53,54].

To maintain analytical clarity and focus, this study specifically concentrated on PM_2.5_ due to PM_2.5_’s well-established relationship with adverse pregnancy outcomes such as preterm birth and LBW, as supported by epidemiological studies [12,26,27]. PM_2.5_ is widely acknowledged as a significant component of urban air pollution, particularly in industrial areas and regions with agricultural burning practices. While other pollutants, including PM_10_, carbon monoxide, and ozone, have also been linked to preterm birth and LBW, incorporating multiple pollutants could potentially lead to multivariable regression complications, making the interpretation of individual pollutant impacts more complex [55]. It is recommended that future research develop multi-pollutant models that could explore cumulative or synergistic effects, potentially utilizing advanced analytical techniques such as machine learning [56].

This study emphasizes the significant public health impact of daily PM_2.5_ exposure during pregnancy. The association between PM_2.5_ concentrations exceeding 31.2 μg/m^3^ and adverse pregnancy outcomes underscores the need for stricter air quality standards. Public health campaigns should raise awareness about risks of PM_2.5_ exposure and about protective measures [57]. Developing surveillance and early warning systems can enable timely protective actions. A multi-sectoral approach integrating environmental and urban planning policies is crucial for reducing PM_2.5_ emissions.

The strengths of this study, which enhance the reliability of our conclusions, include a large sample size, high ethnic homogeneity among participants recruited from the same geographical areas, and a robust exposure assessment utilizing data from two air quality monitoring sites to ensure comprehensive coverage.

Limitations of this study include reliance on population-level PM_2.5_ data, which may not capture individual exposure variations; the lack of PM_2.5_ chemical composition analysis; and the absence of data on long-term child health impacts. Additionally, although the sample size is adequate to address the primary objective, it is too small to assess the effects of PM_2.5_ exposure on important but rare outcomes such as fetal death or fetal distress. Additionally, the data do not provide sufficient evidence to determine whether the same concentration of PM_2.5_ exposure has a different effect size at various stages of pregnancy.

Future research should explore these limitations, including long-term effects of PM_2.5_ exposure, the impact of climate change on PM_2.5_ concentrations, and the potential synergistic effects of PM_2.5_ with other pollutants on pregnancy outcome and child development. Employing personal air quality monitors, assessing socioeconomic and environmental variables, analyzing PM_2.5_ constituents, conducting longitudinal studies, and investigating genetic susceptibility and pollutant interactions will deepen our understanding of the effects of PM_2.5_ effects on maternal and child health. Also, assessment of whether PM_2.5_ exposure increases the risk of developing other pregnancy complications, such as PIH, uteroplacental insufficiency, and fetal growth restriction, should be explored. Finally, the correlation between fetal blood concentrations of PM_2.5_ biomarkers and infant outcomes remains a significant knowledge gap requiring further in-depth study.

Our findings, consistent with previous studies conducted in different populations, strengthen the recommendation for pregnant women to minimize PM_2.5_ exposure as much as possible. Furthermore, our evidence may encourage healthcare providers to incorporate this awareness into antenatal care counseling. Finally, these findings specific to Thai population could potentially prompt the development of preventive measures such as a national policy with special consideration for pregnant women.

## 5. Conclusions

The main finding of this study is that PM_2.5_ exposure at levels exceeding 37.5 μg/m^3^ during pregnancy significantly increases the risk of preterm birth and low birth weight, with greater impacts observed in high-risk pregnancies (women over 35 years of age, those with pre-pregnancy BMI < 18.5 kg/m^2^, those with pregnancy-induced hypertension, and nulliparous women). The critical PM_2.5_ exposure threshold ranged from 32.3 (pre-pregnancy BMI < 18.5 kg/m^2^) to 38.4 μg/m^3^ (maternal age > 35 years) for preterm birth and ranged from 31.2 (maternal age > 35 years) to 38.2 μg/m^3^ (nulliparous) for LBW. Accordingly, our findings could be used to raise awareness among pregnant women and healthcare providers about the detrimental effects of PM_2.5_ exposure or air pollution on maternal and infant health, potentially prompting the development of more effective policies and preventive measures. Ultimately, this could contribute to improving the overall quality of life for the general population.

## Figures and Tables

**Table 1:** Descriptive characteristics of pregnant women and infants, stratified by PM_2.5_ exposure levels during 2016–2022.

Characteristics	Total	PM_2.5_ ≤ 15.0 µg/m^3 A^	PM_2.5_ 15.1–37.5 µg/m^3^	PM_2.5_ > 37.5 µg/m^3^	*p* ^C^
***n***	16,965	445 (2.6%)	15,170 (89.4%)	1350 (8.0%)	
**Maternal Characteristics**					
**Median age (years)**	29.0 (25.0–33.0)	30.0 (26.0–34.0)	29.0 (25.0–33.0)	29.0 (25.0–33.0)	
**Age group**					
<20 years	686 (4.1%)	7 (1.6%)	624 (4.1%)	55 (4.1%)	**0.019**
20–35 years	13,813 (81.4%)	356 (80.0%)	12,356 (81.5%)	1101 (81.6%)	
>35 years	2466 (14.5%)	82 (18.4%)	2190 (14.4%)	194 (14.4%)	
**Median pre-pregnancy BMI (kg/m^2^)**	21.9 (19.7–24.9)	21.9 (19.6,25.5)	21.9 (19.7, 24.9)	21.5 (19.5, 24.4)	
**Classification of BMI** **^B^**					
Underweight (<18.5)	2302 (13.6%)	69 (15.5%)	2023 (13.3%)	210 (15.5%)	
Normal weight (18.5– 22.9)	7919 (46.7%)	188 (42.3%)	7086 (46.7%)	645 (47.8%)	
Overweight (23.0–24.9)	2668 (15.7%)	67 (15.0%)	2388 (15.8%)	213 (15.8%)	**0.015**
Obese (≥25.0)	4076 (24.0%)	121 (27.2%)	3673 (24.2%)	282 (20.9%)	
**Occupation**					
Outdoor	3813 (22.5%)	110 (24.7%)	3380 (22.3%)	323 (23.9%)	
Indoor	8955 (52.8%)	209 (47.0%)	8017 (52.9%)	729 (54.0%)	**0.017**
Jobless	4197 (24.7%)	126 (28.3%)	3773 (24.8%)	298 (22.1%)	
**Education**					
No education or lower than secondary school	3106 (18.3%)	98 (22.0%)	2754 (18.2%)	254 (18.8%)	0.547
Secondary school or higher	13,859 (81.7%)	347 (78.0%)	12,416 (81.8%)	1096 (81.2%)	
**Median frequency of** **antenatal care visits**	8 (6,11)	8 (6,10)	8 (6,11)	7 (6,10)	
**Antenatal care visits**					
<8 visits	6848 (40.4%)	189 (42.5%)	5971 (39.4%)	688 (51.0%)	**<0.001**
≥8 visits	10,117 (59.6%)	256 (57.5%)	9199 (60.6%)	662 (49.0%)	
**Parity**					
Nulliparous women	9138 (53.9%)	227 (51.0%)	8214 (54.2%)	697 (51.6%)	0.097
Parous women	7827 (46.1%)	218 (49.0%)	6956 (45.8%)	653 (48.4%)	
**History of abortion**					
None	13,174 (77.7%)	335 (75.3%)	11,804 77.8%)	1035 (76.7%)	0.298
Once or more	3791 (22.3%)	110 (24.7%)	3366 (22.2%)	315 (23.3%)	
**Complications**					
Pregnancy-induced hypertension	1481 (8.7%)	57 (12.8%)	1280 (8.4%)	144 (10.7%)	**<0.001**
Gestational diabetes	2401 (14.2%)	77 (17.3%)	2130 (14.0%)	194 (14.4%)	0.146
Thalassemia	2866 (16.9%)	62 (13.9%)	2568 (16.9%)	236 (17.5%)	0.210
Hepatitis B virus	717 (4.2%)	15 (3.4%)	652 (4.3%)	50 (3.7%)	0.385
Human immunodeficiency virus	133 (0.8%)	1 (0.22%)	122 (0.8%)	10 (0.7%)	0.802
Syphilis	207 (1.2%)	15 (3.4%)	175 (1.2%)	17 (1.3%)	0.542
Condyloma	24 (0.1%)	1 (0.2%)	20 (0.1%)	3 (0.2%)	0.625
Epilepsy	18 (0.1%)	2 (0.5%)	13 (0.1%)	3 (0.2%)	0.210
**Infant Characteristics**					
Median gestational age at delivery (weeks)	38 (38, 39)	38 (36,39)	39 (38, 39)	38 (37, 39)	**<0.001 ^D^**
Median birthweight (grams)	3065 (2785, 3350)	2950 (2470, 3240)	3075 (2800, 3355)	2980 (2650, 3290)	**<0.001 ^D^**
**APGAR score at 5 min**					
<7/10	256 (1.5%)	19 (4.3%)	207 (1.4%)	30 (2.2%)	**<0.001**
≥7/10	16,709 (98.5%)	426 (95.7%)	14,963 (98.6%)	1320 (97.8%)	
**Infant sex**					
Male	8624 (50.8%)	211 (47.4%)	7692 (50.7%)	721 (53.4%)	0.057
Female	8341 (49.2%)	234 (52.6%)	7478 (49.3%)	629 (46.6%)	
**Mode of delivery**					
Vaginal delivery	11,812 (69.6%)	273 (61.3%)	10,600 (69.9%)	939(69.6%)	**<0.001**
Cesarian section	5153 (30.4%)	172 (38.7%)	4570 (30.1%)	411 (30.4%)	
**Hospital sites**					
Maharaj Nakorn Chiang Mai hospital	8420 (49.6%)	259 (58.2%)	7495 (49.4%)	666 (49.3%)	**0.001**
Regional Health Promotion Center1 Hospital, Chiang Mai	8545 (50.4%)	186 (41.8%)	7675 (50.6%)	684 (50.7%)	

Continuous data are reported as median (IQR: quartile 1, quartile 3) and categorical data as *n* (%). PM_2.5_, particulate matter 2.5 μm or less in diameter; APGAR: appearance, pulse, grimace, activity, respiration. ^A^ PM_2.5_ concentration thresholds were categorized into three groups: ≤15.0 µg/m^3^, 15.1–37.5 µg/m^3^, and >37.5 µg/m^3^. ^B^ BMI classification based on the Asia–Pacific criteria. ^C^ Derived from chi-square or Fisher’s exact test as appropriate. ^D^ Kruskal–Wallis test compared the three study conditions. The *p*-values for statistically significant overall differences between groups (*p* < 0.05) are shown in bold.

**Table 2:** Average daily PM_2.5_ concentrations for a range of adverse pregnancy outcomes according to the timing of exposure.

Variable	Preterm Birth ^A^	Term Birth	LBW ^B^	NBW	SGA ^C^	Non-SGA
*n* (%)	1780 (10.5%)	15,185 (89.5%)	1894 (11.2%)	15,071 (88.8%)	1361 (8.0%)	15,604 (92.0%)
**PM_2.5_** **Exposure Concentrations ^D^**						
1st trimester						
Median (IQR)	22.3 (14.4, 39.5)	22.0 (14.4, 40.1)	21.8 (14.4, 39.5)	22.1 (14.4, 40.1)	21.3 (14.5, 39.3)	22.1 (14.4, 40.1)
Range	7.9–70.4	8.1–70.4	7.9–70.4	8.1–70.4	8.1–70.4	7.9–70.4
2nd trimester						
Median (IQR)	20.2 (13.7, 37.0)	20.8 (13.7, 37.1)	20.5 (13.6, 37.3)	20.4 (13.7, 37.0)	20.7 (13.5, 36.6)	20.4 (13.8, 37.1)
Range	8.1–70.4	8.1–70.4	8.1–70.4	8.1–70.4	8.1–70.4	8.1–70.4
3rd trimester						
Median (IQR)	19.4 (12.8, 34.4)	19.9 (13.2, 35.8)	19.4 (12.7, 34.8)	19.9 (13.2, 3)	19.9 (12.7, 37.0)	19.8 (13.1, 35.5)
Range	5.1–118.9	7.6–73.6	5.1–118.9	7.3–78.3	5.1–103.5	6.2–118.9
Entire pregnancy						
Median (IQR)	26.5 (19.5, 33.9)	27.1 (21.0, 32.3)	26.9 (20.0, 33.6)	27.1 (20.9, 32.3)	26.9 (20.8, 32.3)	27.1 (20.9, 32.5)
Range	9.5–52.9	12.5–40.3	9.5–52.9	12.3–41.8	10.5–46.6	9.5–52.9

Continuous data are reported as median (IQR: quartile 1, quartile 3) and categorical data as *n* (%). PM_2.5_ (particulate matter 2.5 μm or less in diameter), LBW (low birth weight), NBW (normal birth weight), SGA (small for gestational age), non-SGA (appropriate for gestational age and large for gestational age). ^A^ Gestational age expressed in complete weeks. ^B^ Birth weight expressed in grams. ^C^ Birth weight centile calculated using INTERGROWTH-21st standards. ^D^ PM_2.5_ exposure concentrations are expressed as median (IQR: quartile 1, quartile 3) in micrograms per cubic meter (μg/m^3^), and ranges represent the minimum and maximum daily concentrations. These values are calculated based on average daily concentrations during each trimester and the entire pregnancy, using data from air quality monitoring stations.

**Table 3:** Adjusted odds ratios (95% confidence intervals) of preterm birth, low birth weight, and small for gestational age by PM_2.5_ concentration categories during different time periods of pregnancy.

Pregnancy Period	PM_2.5_ Category (μg/m^3^)	Preterm Birth		LBW		SGA	
		aOR (95% CI)	*p* ^A^	aOR (95% CI)	*p* ^A^	aOR (95% CI)	*p* ^A^
First trimester	≤15.0	1.05 (0.93, 1.19)	0.417	1.04 (0.92, 1.17)	0.546	1.00 (0.88, 1.15)	0.965
	15.1–37.5	Ref		Ref		Ref	
	>37.5	0.99 (0.87, 1.11)	0.811	0.98 (0.87, 1.10)	0.735	0.95 (0.83, 1.09)	0.466
Second trimester	≤15.0	0.98 (0.87,1.11)	0.779	0.99 (0.88,1.11)	0.889	1.02 (0.90, 1.17)	0.718
	15.1–37.5	Ref		Ref		Ref	
	>37.5	1.02 (0.90, 1.16)	0.702	1.06 (0.94, 1.20)	0.314	0.96 (0.83,1.10)	0.550
Third trimester	≤15.0	1.04 (0.92, 1.16)	0.552	1.04 (0.93, 1.17)	0.470	1.09 (0.96, 1.24)	0.204
	15.1–37.5	Ref		Ref		Ref	
	>37.5	1.05 (0.93, 1.20)	0.420	1.13 (0.99, 1.27)	0.055	1.11 (0.97, 1.28)	0.141
Entire pregnancy	≤15.0	3.18 (2.52, 4.01)	**<0.001**	3.02 (2.41, 3.80)	**<0.001**	1.07 (0.76, 1.51)	0.696
	15.1–37.5	Ref		Ref		Ref	
	>37.5	2.19 (1.89, 2.55)	**<0.001**	1.99 (1.71, 2.31)	**<0.001**	1.04 (0.85, 1.28)	0.673

The adjusted odds ratios (ORs) were adjusted for potential confounders, including maternal age, pre-pregnancy BMI, frequency of antenatal care visits, parity, pregnancy-induced hypertension, thalassemia, history of abortion, infant sex, gestational diabetes, maternal occupation, and maternal education level. aOR (adjusted odds ratio), CI (confidence interval), LBW (low birth weight), SGA (small for gestational age). ^A^ Results derived from multivariable logistic regression models, with PM_2.5_ 15.1–37.5 μg/m^3^ as reference category. Statistically significant differences (*p* < 0.05) are indicated in bold.

**Table 4:** Odds ratios (95% confidence intervals) of preterm birth and low birth weight per 10 μg/m^3^ increase in PM_2.5_ concentration during the entire pregnancy, stratified by potential confounders.

Pregnancy Period	Categories	N	Preterm Birth		LBW	
			aOR (95% CI)	*p* ^A^	aOR (95% CI)	*p* ^A^
Maternal age (years)	≤35.0	14,136	1.07 (0.98, 1.16)	0.125	1.06 (0.98, 1.02)	0.173
	>35.0	2384	1.17 (0.98, 1.39)	0.069	1.35 (1.14, 159)	**0.001**
Pre-pregnancy BMI (kg/m^2^)	<18.5	14,287	1.26 (1.05, 1.50)	**0.016**	1.14 (0.697, 1.33)	0.122
	≥18.5	2233	1.06 (0.98, 1.15)	0.173	1.14 (0.98, 1.34)	0.098
Pregnancy-induced hypertension	Yes	1424	1.09 (1.01,1.19)	**0.030**	1.11 (1.02, 1.20)	**0.012**
	No	15,096	1.12 (0.91, 1.37)	0.276	1.19 (0.97, 1.46)	0.089
Parity	Nulliparous	9811	1.12 (1.02, 1.24)	**0.023**	1.15 (1.02, 1.29)	**0.022**
	Parous	7609	1.04 (0.93, 1.17)	0.458	1.09 (0.99, 1.20)	0.055

Adjusted ORs are presented per 10 μg/m^3^ increment in PM_2.5_ concentration during the entire pregnancy. Adjustments were made by potential confounders, including maternal age, pre-pregnancy BMI, frequency of antenatal care visits, parity, pregnancy-induced hypertension, thalassemia, history of abortion, infant sex, gestational diabetes, maternal occupation, and maternal education level. aOR (adjusted odds ratio), CI (confidence interval), LBW (low birth weight), BMI (body mass index). ^A^ Derived from multivariable logistic regression models; statistically significant differences (*p* < 0.05) are indicated in bold.

## Data Availability

The data supporting this study is available within the article. Raw data supporting this study’s findings are available from the corresponding author upon reasonable request.

## Supplementary Materials

The following supporting information can be downloaded at: https://www.mdpi.com/article/10.3390/toxics13040304/s1, Figure S1: Map of the study area in Chiang Mai City, Thailand. Figure S2: Daily average variation in PM_2.5_ concentration of particulate matters from 1 January 2015 to 31 December 2022 over Chiang Mai, Thailand. Figure S3: Annual distribution of PM_2.5_ concentrations (µg/m^3^) from 2015 to 2022. Table S1: Univariate analysis of risk factors for preterm birth, low birth weight, and small for gestational age using logistic regression.

## References

[1] Gogna P., Borghese M.M., Villeneuve P.J., Kumarathasan P., Johnson M., Shutt R.H., Ashley-Martin J., Bouchard M.F., King W.D. (2024). A cohort study of the multipollutant effects of PM_2.5_, NO_2_, and O_3_ on C-reactive protein levels during pregnancy. Environ. Epidemiol..

[2] Shen Y., Wang Y., Fu Z., Zhou T., Yuan Z., Gao J., Ji Y. (2024). Ambient PM_2.5_, household environment and preterm birth: A birth cohort study in Shandong, China. Atmos. Environ..

[3] Jiang P., Li Y., Tong M.K., Ha S., Gaw E., Nie J., Mendola P., Wang M. (2024). Wildfire particulate exposure and risks of preterm birth and low birth weight in the Southwestern United States. Public Health.

[4] Lin W., Lai Y., Zhuang S., Wei Q., Zhang H., Hu Q., Cheng P., Zhang M., Zhai Y., Wang Q. (2023). The effects of prenatal PM_2.5_ oxidative potential exposure on feto-placental vascular resistance and fetal weight: A repeated-measures study. Environ. Res..

[5] Li S., Li L., Zhang C., Fu H., Yu S., Zhou M., Guo J., Fang Z., Li A., Zhao M. (2023). PM_2.5_ leads to adverse pregnancy outcomes by inducing trophoblast oxidative stress and mitochondrial apoptosis via KLF9/CYP1A1 transcriptional axis. eLife.

[6] Dadvand P., Basagaña X., Figueras F., Martinez D., Beelen R., Cirach M., de Nazelle A., Hoek G., Ostro B., Nieuwenhuijsen M.J. (2014). Air pollution and preterm premature rupture of membranes: A spatiotemporal analysis. Am. J. Epidemiol..

[7] Collins A., Weitkamp J.H., Wynn J.L. (2018). Why are preterm newborns at increased risk of infection?. Arch. Dis. Child.-Fetal Neonatal Ed..

[8] Siffel C., Hirst A., Sarda S., Kuzniewicz M., Li D.-K. (2022). The clinical burden of extremely preterm birth in a large medical records database in the United States: Mortality and survival associated with selected complications. Early Hum. Dev..

[9] Zhang X., Fan C., Ren Z., Feng H., Zuo S., Hao J., Liao J., Zou Y., Ma L. (2020). Maternal PM_2.5_ exposure triggers preterm birth: A cross-sectional study in Wuhan, China. Glob. Health Res. Policy.

[10] Park S., Kwon E., Lee G., You Y.A., Kim S.M., Hur Y.M., Jung S., Jee Y., Park M.H., Na S.H. (2023). Effect of Particulate Matter 2.5 on Fetal Growth in Male and Preterm Infants through Oxidative Stress. Antioxidants.

[11] Wang X., Wang X., Gao C., Xu X., Li L., Liu Y., Li Z., Xia Y., Fang X. (2023). Relationship Between Outdoor Air Pollutant Exposure and Premature Delivery in China-Systematic Review and Meta-Analysis. Int. J. Public Health.

[12] Li L., Zhang X. (2024). The causal impact of fetal exposure to PM_2.5_ on birth outcomes: Evidence from rural China. Econ. Hum. Biol..

[13] Grillo M.A., Mariani G., Ferraris J.R. (2021). Prematurity and Low Birth Weight in Neonates as a Risk Factor for Obesity, Hypertension, and Chronic Kidney Disease in Pediatric and Adult Age. Front. Med..

[14] Zhu Z., Hu H., Benmarhnia T., Ren Z., Luo J., Zhao W., Chen S., Wu K., Zhang X., Wang L. (2022). Gestational PM_2.5_ exposure may increase the risk of small for gestational age through maternal blood pressure and hemoglobin: A mediation analysis based on a prospective cohort in China, 2014–2018. Ecotoxicol. Environ. Saf..

[15] Ludvigsson J.F., Lu D., Hammarström L., Cnattingius S., Fang F. (2018). Small for gestational age and risk of childhood mortality: A Swedish population study. PLoS Med..

[16] Zhu N., Ji X., Geng X., Yue H., Li G., Sang N. (2021). Maternal PM_2.5_ exposure and abnormal placental nutrient transport. Ecotoxicol. Environ. Saf..

[17] Ardiyani V., Wooster M., Grosvenor M., Lestari P., Suri W. (2023). The infiltration of wildfire smoke and its potential dose on pregnant women: Lessons learned from Indonesia wildfires in 2019. Heliyon.

[18] Ho T.H., Van Dang C., Pham T.T.B., Thi Hien T., Wangwongwatana S. (2023). Ambient particulate matter (PM_2.5_) and adverse birth outcomes in Ho Chi Minh City, Vietnam. Hyg. Environ. Health Adv..

[19] Ponsawansong P., Prapamontol T., Rerkasem K., Chantara S., Tantrakarnapa K., Kawichai S., Li G., Fang C., Pan X., Zhang Y. (2023). Sources of PM_2.5_ Oxidative Potential During Haze and Non-Haze Seasons in Chiang Mai, Thailand. Aerosol Air Qual. Res..

[20] Kawichai S., Prapamontol T., Cao F., Song W., Zhang Y.-L. (2024). Characteristics of Carbonaceous Species of PM_2.5_ in Chiang Mai City, Thailand. Aerosol Air Qual. Res..

[21] Gazette R. Ministry of Public Health Announcement on General Air Quality Standards. https://ratchakitcha.soc.go.th/documents/140D157S0000000000300.pdf.

[22] WHO WHO Global Air Quality Guidelines: Particulate Matter (PM2.5 and PM10), Ozone, Nitrogen Dioxide, Sulfur Dioxide and Carbon Monoxide. https://iris.who.int/bitstream/handle/10665/345329/9789240034228-eng.pdf?sequence=1.

[23] Kawichai S., Sripan P., Rerkasem A., Rerkasem K., Srisukkham W. (2025). Long-term retrospective predicted concentration of PM_2.5_ in upper northern Thailand using machine learning models. Toxics.

[24] Villar J., Cheikh Ismail L., Victora C.G., Ohuma E.O., Bertino E., Altman D.G., Lambert A., Papageorghiou A.T., Carvalho M., Jaffer Y.A. (2014). International standards for newborn weight, length, and head circumference by gestational age and sex: The Newborn Cross-Sectional Study of the INTERGROWTH-21st Project. Lancet.

[25] Bachwenkizi J., Liu C., Meng X., Zhang L., Wang W., van Donkelaar A., Martin R.V., Hammer M.S., Chen R., Kan H. (2022). Maternal exposure to fine particulate matter and preterm birth and low birth weight in Africa. Environ. Int..

[26] Parasin N., Amnuaylojaroen T., Saokaew S. (2024). Prenatal PM_2.5_ Exposure and Its Association with Low Birth Weight: A Systematic Review and Meta-Analysis. Toxics.

[27] Ghosh R., Causey K., Burkart K., Wozniak S., Cohen A., Brauer M. (2021). Ambient and household PM_2.5_ pollution and adverse perinatal outcomes: A meta-regression and analysis of attributable global burden for 204 countries and territories. PLoS Med..

[28] Mueller W., Tantrakarnapa K., Johnston H.J., Loh M., Steinle S., Vardoulakis S., Cherrie J.W. (2021). Exposure to ambient particulate matter and biomass burning during pregnancy: Associations with birth weight in Thailand. J. Expo. Sci. Environ. Epidemiol..

[29] Bekkar B., Pacheco S., Basu R., DeNicola N. (2020). Association of Air Pollution and Heat Exposure with Preterm Birth, Low Birth Weight, and Stillbirth in the US: A Systematic Review. JAMA Netw. Open.

[30] Sun X., Luo X., Zhao C., Chung Ng R.W., Lim C.E., Zhang B., Liu T. (2015). The association between fine particulate matter exposure during pregnancy and preterm birth: A meta-analysis. BMC Pregnancy Childbirth.

[31] Pedersen M., Giorgis-Allemand L., Bernard C., Aguilera I., Andersen A.M., Ballester F., Beelen R.M., Chatzi L., Cirach M., Danileviciute A. (2013). Ambient air pollution and low birthweight: A European cohort study (ESCAPE). Lancet Respir. Med..

[32] Rich D.Q., Liu K., Zhang J., Thurston S.W., Stevens T.P., Pan Y., Kane C., Weinberger B., Ohman-Strickland P., Woodruff T.J. (2015). Differences in Birth Weight Associated with the 2008 Beijing Olympics Air Pollution Reduction: Results from a Natural Experiment. Environ. Health Perspect..

[33] O’Sharkey K., Xu Y., Cabison J., Rosales M., Yang T., Chavez T., Johnson M., Lerner D., Lurvey N., Corral C.M.T. (2023). Effects of in-utero personal exposure to PM_2.5_ sources and components on birthweight. Sci. Rep..

[34] Cai J., Zhao Y., Kan J., Chen R., Martin R., van Donkelaar A., Ao J., Zhang J., Kan H., Hua J. (2020). Prenatal Exposure to Specific PM_2.5_ Chemical Constituents and Preterm Birth in China: A Nationwide Cohort Study. Environ. Sci. Technol..

[35] Tapia V.L., Vasquez B.V., Vu B., Liu Y., Steenland K., Gonzales G.F. (2020). Association between maternal exposure to particulate matter PM_2.5_ and adverse pregnancy outcomes in Lima, Peru. J. Expo. Sci. Environ. Epidemiol..

[36] Zhu X., Liu Y., Chen Y., Yao C., Che Z., Cao J. (2015). Maternal exposure to fine particulate matter (PM_2.5_) and pregnancy outcomes: A meta-analysis. Environ. Sci. Pollut. Res. Int..

[37] Guan T., Xue T., Gao S., Hu M., Liu X., Qiu X., Liu X., Zhu T. (2019). Acute and chronic effects of ambient fine particulate matter on preterm births in Beijing, China: A time-series model. Sci. Total Environ..

[38] Estarlich M., Ballester F., Aguilera I., Fernández-Somoano A., Lertxundi A., Llop S., Freire C., Tardón A., Basterrechea M., Sunyer J. (2011). Residential exposure to outdoor air pollution during pregnancy and anthropometric measures at birth in a multicenter cohort in Spain. Environ. Health Perspect..

[39] Li J., Yan J., Jiang W. (2023). The role of maternal age on adverse pregnancy outcomes among primiparous women with singleton birth: A retrospective cohort study in urban areas of China. J. Matern. Fetal Neonatal Med..

[40] Du H., Sun Y., Zhang Y., Wang S., Zhu H., Chen S., Pan H. (2022). Interaction of PM_2.5_ and pre-pregnancy body mass index on birth weight: A nationwide prospective cohort study. Front. Endocrinol..

[41] Zhang M., Mueller N.T., Wang H., Hong X., Appel L.J., Wang X. (2018). Maternal Exposure to Ambient Particulate Matter ≤ 2.5 µm During Pregnancy and the Risk for High Blood Pressure in Childhood. Hypertension.

[42] Ahn T.G., Kim Y.J., Lee G., You Y.A., Kim S.M., Chae R., Hur Y.M., Park M.H., Bae J.G., Lee S.J. (2024). Association Between Individual Air Pollution (PM_10_, PM_2.5_) Exposure and Adverse Pregnancy Outcomes in Korea: A Multicenter Prospective Cohort, Air Pollution on Pregnancy Outcome (APPO) Study. J. Korean Med. Sci..

[43] Fleischer N.L., Merialdi M., van Donkelaar A., Vadillo-Ortega F., Martin R.V., Betran A.P., Souza J.P. (2014). Outdoor air pollution, preterm birth, and low birth weight: Analysis of the world health organization global survey on maternal and perinatal health. Environ. Health Perspect..

[44] Zhang B., Gong X., Han B., Chu M., Gong C., Yang J., Chen L., Wang J., Bai Z., Zhang Y. (2022). Ambient PM_2.5_ exposures and systemic inflammation in women with early pregnancy. Sci. Total Environ..

[45] Niu B.Y., Li W.K., Li J.S., Hong Q.H., Khodahemmati S., Gao J.F., Zhou Z.X. (2020). Effects of DNA Damage and Oxidative Stress in Human Bronchial Epithelial Cells Exposed to PM_2.5_ from Beijing, China, in Winter. Int. J. Environ. Res. Public Health.

[46] Meng Q., Liu J., Shen J., Del Rosario I., Janzen C., Devaskar S.U., Lakey P.S.J., Shiraiwa M., Weichenthal S., Zhu Y. (2024). Ambient exposure to fine particulate matter with oxidative potential affects oxidative stress biomarkers in pregnancy. Am. J. Epidemiol..

[47] Zhou J., Yu S., Wang C., Fu R., Wu D., Abuduwaili D., Wang C. (2024). Relationship between environmental PM_2.5_ exposure in early pregnancy and thyroid hormone levels in pregnant women. Ecotoxicol. Environ. Saf..

[48] Rider C.F., Carlsten C. (2019). Air pollution and DNA methylation: Effects of exposure in humans. Clin. Epigenetics.

[49] Breton C.V., Marsit C.J., Faustman E., Nadeau K., Goodrich J.M., Dolinoy D.C., Herbstman J., Holland N., LaSalle J.M., Schmidt R. (2017). Small-Magnitude Effect Sizes in Epigenetic End Points are Important in Children’s Environmental Health Studies: The Children’s Environmental Health and Disease Prevention Research Center’s Epigenetics Working Group. Environ. Health Perspect..

[50] Fouladi F., Bailey M.J., Patterson W.B., Sioda M., Blakley I.C., Fodor A.A., Jones R.B., Chen Z., Kim J.S., Lurmann F. (2020). Air pollution exposure is associated with the gut microbiome as revealed by shotgun metagenomic sequencing. Environ. Int..

[51] Jarernwong K., Gheewala S.H., Sampattagul S. (2023). Health Impact Related to Ambient Particulate Matter Exposure as a Spatial Health Risk Map Case Study in Chiang Mai, Thailand. Atmosphere.

[52] Ming X., Yang Y., Li Y., He Z., Tian X., Cheng J., Zhou W. (2024). Association between risk of preterm birth and long-term and short-term exposure to ambient carbon monoxide during pregnancy in chongqing, China: A study from 2016–2020. BMC Public Health.

[53] Fussell J.C., Jauniaux E., Smith R.B., Burton G.J. (2024). Ambient air pollution and adverse birth outcomes: A review of underlying mechanisms. BJOG.

[54] Wang Q., Miao H., Warren J.L., Ren M., Benmarhnia T., Knibbs L.D., Zhang H., Zhao Q., Huang C. (2021). Association of maternal ozone exposure with term low birth weight and susceptible window identification. Environ. Int..

[55] Evangelopoulos D., Katsouyanni K., Schwartz J., Walton H. (2021). Quantifying the short-term effects of air pollution on health in the presence of exposure measurement error: A simulation study of multi-pollutant model results. Environ. Health.

[56] Wang S., Zhang T., Li Z., Hong J. (2024). Exploring pollutant joint effects in disease through interpretable machine learning. J. Hazard. Mater..

[57] Ramírez A.S., Ramondt S., Van Bogart K., Perez-Zuniga R. (2019). Public Awareness of Air Pollution and Health Threats: Challenges and Opportunities for Communication Strategies to Improve Environmental Health Literacy. J. Health Commun..

