# Supplementary material for: Association of Maternal PM2.5 Exposure with Preterm Birth and Low Birth Weight: A Large-Scale Cohort Study in Northern Thailand (2016–2022)
Source: Toxics. 2025 Apr 13;13(4):304. doi: 10.3390/toxics13040304 (PMC12031216; doi:10.3390/toxics13040304)
Supplement: Supplementary file 1 [file toxics-13-00304-s001.zip › toxics-3462766-supplementary.pdf]

Supplementary data

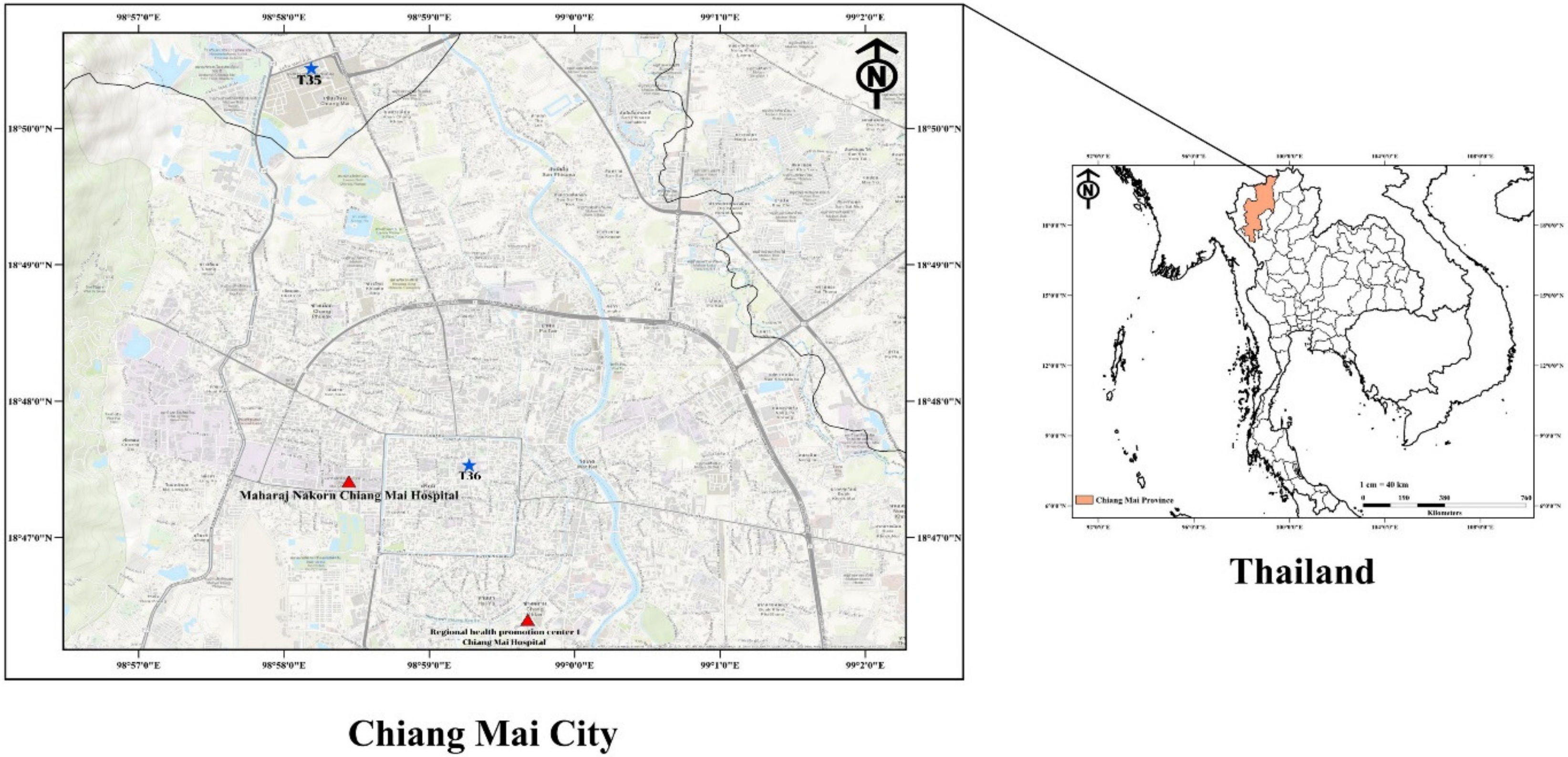

Supplementary Figure S1. Map of the study area in Chiang Mai City, Thailand.

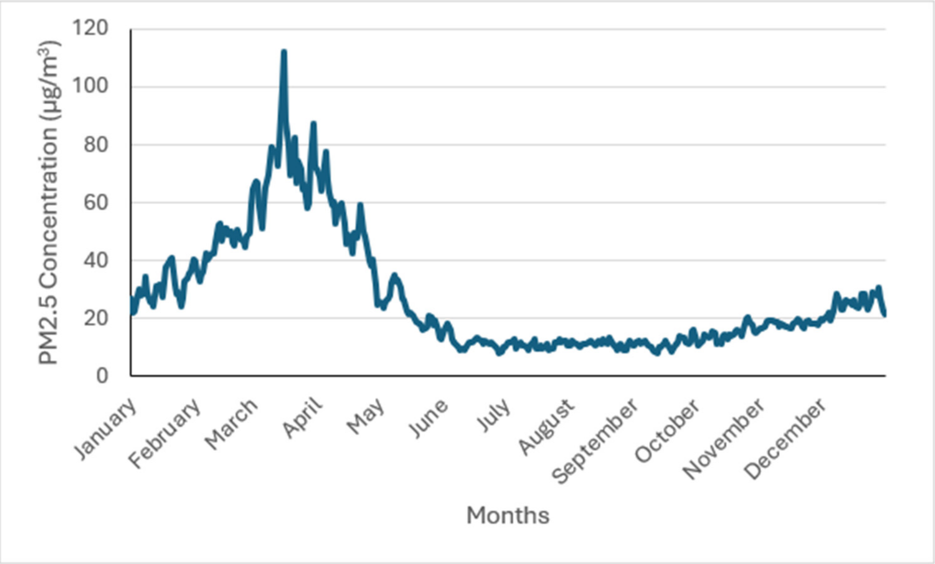

Supplementary Figure S2. Daily average variation in $PM_{2.5}$ concentration of particulate matters from 1 January 2015 to 31 December 2022 over Chiang Mai, Thailand.

From 2015 to 2022, $PM_{2.5}$ concentrations in Muang District, Chiang Mai, exhibited clear seasonal variation, with an annual average of 27.2 µg/m³, exceeding the standard level (15 µg/m³). Concentration of $PM_{2.5}$ were high in the dry season (December to May), peaking in March at 63.5 µg/m³ (IQR: 45.7, 89.1), with a recorded maximum of 112 µg/m³. In contrast, the lowest levels were observed in July, at 10.6 µg/m³ (IQR: 7.6, 13.1). $PM_{2.5}$ concentrations during the rainy season (June to November) were consistently low.

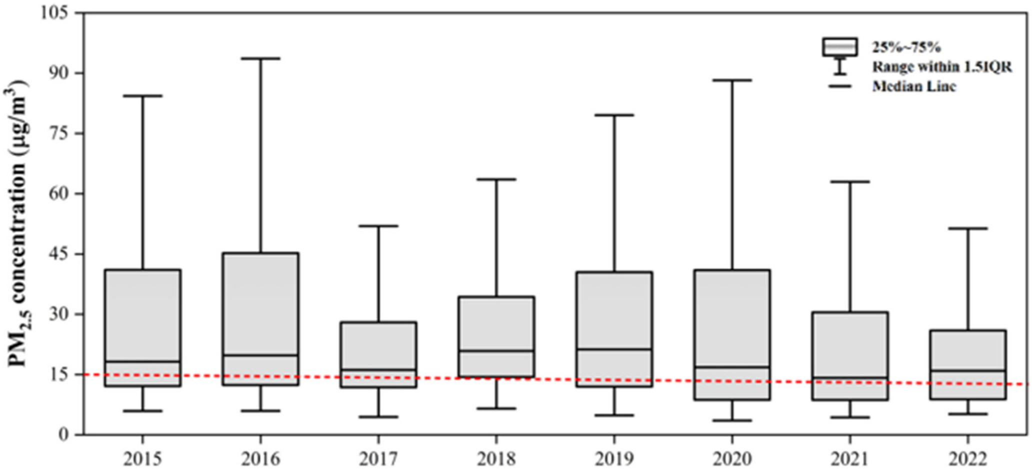

Supplementary Figure S3. Annual distribution of $PM_{2.5}$ concentrations (µg/m³) from 2015 to 2022.

Supplementary Table S1. Univariate Analysis of Risk Factors for Preterm Birth, Low Birth Weight, and Small for Gestational Age Using Logistic Regression.

Variable	Preterm birth		LBW		SGA	
	**OR (95 % CI)**	***p*** **[C]**	**OR (95 % CI)**	***p*** **[C]**	**OR (95 % CI)**	***p*** **[C]**
**Maternal Characteristics**						
**Age(years)**	1.27 (1.19, 1.35)	**<0.001**	1.27 (1.20, 1.35)	**<0.001**	1.05 (0.98, 1.14)	0.163
<20 years	1.61 (1.29, 2.00)	**<0.001**	1.62 (1.31, 2.01)	**<0.001**	1.40 (1.09,1.80)	**0.009**
20-35 years	Ref		Ref		Ref	
>35 years	1.58 (1.39, 1.80)	**<0.001**	1.58 (1.40, 1.79)	**<0.001**	1.07 (0.91, 1.25)	0.423
**Pre-pregnancy Body mass index (kg/m²) [A]**	0.96 (0.94, 0.97)	**<0.001**	0.93 (0.92, 0.94)	**<0.001**	0.93 (0.92,0.94)	**<0.001**
Underweight (<18.5)	1.48 (1.29, 1.70)	**<0.001**	1.79 (1.58, 2.03)	**<0.001**	1.68 (1.45, 1.94)	**<0.001**
Normal weight (18.5-22.9)	Ref		Ref		Ref	
Overweight (23.0-24.9)	1.02 (0.89, 1.18)	0.748	0.87 (0.76, 1.01)	0.352	0.74 (0.62,0.88)	**0.001**
Obese (>25.0)	0.89 (0.79, 1.02)	0.089	0.75 (0.66, 0.86)	**<0.001**	0.70 (0.60,0.81)	**<0.001**
**Occupation**						
Outdoor	1.17 (1.04, 1.32)	**0.011**	1.21 (1.08. 1.36)	**0.001**	1.08 (0.94,1.24)	0.278
Indoor	Ref		Ref		Ref	
Jobless	1.08 (0.96, 1.21)	0.222	1.07 (0.95, 1.20)	0.274	1.07 (0.94,1.22)	0.327
**Education**						
No education or lower than secondary school	1.22 (1.08, 1.38)	**0.001**	1.00 (0.89,1.14)	0.938	0.95 (0.82,1.10)	0.503
Secondary school or higher	Ref		Ref		Ref	
**Antenatal care visits**	0.82(0.81,0.83)	**<0.001**	0.87 (0.86, 0.88)	**<0.001**	1.00(0.99,1.02)	0.562
<8 visits	Ref		Ref		Ref	
≥8 visits	0.35 (0.32, 0.39)	**<0.001**	0.49 (0.44, 0.54)	**<0.001**	1.07(0.95,1.20)	0.264
**Parity**	0.97 (0.91, 1.04)	0.439	0.81 (0.75, 0.87	**<0.001**	0.59 (0.54,0.65)	**<0.001**
Nulliparous women	Ref		Ref		Ref	
Parous women	0.89 (0.80, 0.98)	**0.018**	0.70 (0.64, 0.78)	**<0.001**	0.51(0.45,0.57)	**<0.001**
**History of abortion**						
None	Ref		Ref		Ref	
Once or more	1.12 (0.99, 1.25)	0.060	1.12 (1.01, 1.26)	**0.042**	0.94(0.82,1.08)	0.373
**Complications**						

Pregnancy-induced hypertension	1.96 (1.69. 2.26)	**<0.001**	1.98 (1.72, 2.28)	**<0.001**	1.54 (1.30,1.83)	**<0.001**
Gestational Diabetes	1.12 (0.98, 1.29)	0.097	1.21 (1.06, 1.38)	**0.004**	1.00 (0.86, 1.18)	0.975
Thalassemia	0.55 (0.47, 0.65)	**<0.001**	0.52 (0.45, 0.61)	**<0.001**	0.97 (0.83, 1.12)	0.655
Hepatitis B virus	0.98 (0.77, 1.25)	0.878	0.87 (0.68, 1.12)	0.273	0.69 (0.50, 0.94)	**0.021**
Human Immunodeficiency Virus	0.92 (0.52, 1.64)	0.786	1.41 (0.88, 2.28)	0.156	1.46 (0.85, 2.51)	0.168
Syphilis	0.91 (0.57, 1.45)	0.695	1.41 (0.96, 2.07)	0.081	1.59 (1.04,2.42)	**0.032**
Condyloma	1.22 (0.36, 4.09)	0.748	1.14 (0.34, 3.82)	0.835	0.50 (0.07,3.69)	0.495
Epilepsy	Null		1.59 (0.46, 5.50)	0.462	3.28 (1.08 ,9.99)	**0.036**
**Infant Characteristics**						
**APGAR score at 5 minutes**	0.56 (0.53, 0.58)	**<0.001**	0.57 (0.55, 0.60)	**<0.001**	0.872 (0.83, 0.92)	**<0.001**
<7/10	15.7 (12.2, 20.4)	**<0.001**	18.2 (13.9, 23.7)	**<0.001**	2.63 (1.91,3.63)	**<0.001**
≥7/10	Ref		Ref		Ref	
**Infant sex**						
Male	1.11 (1.00, 1.22)	**0.044**	0.86 (0.78, 0.94)	**0.001**	1.12 (1.00,1.25)	0.054
**Mode of delivery**						
Vaginal delivery	Ref		Ref		Ref	
Cesarian section	1.57 (1.42, 1.74)	**<0.001**	1.60 (1.45, 1.76)	**<0.001**	0.95 (0.84,1.07)	0.410
**$PM_{2.5}$ category (μg/m^3) [B]**						
1st trimester						
≤15.0	1.05 (0.93, 1.18)	0.424	1.03 (0.92, 1.15)	0.573	0.99 (0.87, 1.14)	0.938
15.1-37.5	Ref		Ref		Ref	
>37.5	0.99 (0.88, 1.11)	0.871	0.98 (0.87, 1.10)	0.759	0.94 (0.82, 1.08)	0.399
2nd trimester						
≤15.0	1.00 (0.89, 1.12)	0.977	1.01 (0.90, 1.13)	0.875	1.03 (0.91, 1.17)	0.620
15.1-37.5	Ref		Ref		Ref	
>37.5	1.09 (0.97, 1.23)	0.159	1.12 (0.98, 1.26)	0.157	0.98 (0.85, 1.13)	0.770
3rd trimester						
≤15.0	1.05 (0.94, 1.18)	0.387	1.06 (0.95, 1.18)	0.306	1.09 (0.96, 1.24)	0.182
15.1-37.5	Ref		Ref		Ref	
>37.5	1.09 (0.97, 1.24)	0.139	1.09 (0.98, 1.22)	0.118	1.11 (0.97, 1.28)	0.140
Entire pregnancy						
≤15.0	3.36 (2.69, 4.18)	**<0.001**	3.18 (2.55, 3.95)	**<0.001**	1.08 (0.77, 1.51)	0.662
15.1-37.5	Ref		Ref		Ref	
>37.5	2.46 (2.13, 2.85)	**<0.001**	2.17 (1.88, 2.51)	**<0.001**	1.07 (0.87, 1.30)	0.538

OR, odds ratio; CI, confidence interval; LBW, low birth weight; SGA, small for gestational age; Ref, reference category; $PM_{2.5}$: Particulate matter 2.5 micrometers or less in diameter; APGAR: Appearance, Pulse, Grimace, Activity, Respiration.

[A] BMI classification based on the Asia-Pacific criteria

[B] PM2.5 concentration thresholds were categorized into three groups: ≤15.0 μg/m³, 15.1–37.5 μg/m³, and >37.5 μg/m³. The threshold of 37.5 μg/m³.

[C] Derived from univariate logistic regression models; statistically significant differences (p<0.05) are indicated in bold.
